# Comparison of serum zinc concentrations and body antioxidant status between young women with premenstrual syndrome and normal controls: A case-control study

**Published:** 2016-11

**Authors:** Sanaz Fathizadeh, Reza Amani, Mohammad Hossein Haghighizadeh, Razieh Hormozi

**Affiliations:** 1 *Nutrition and Metabolic Disease Research Center, Ahvaz Jundishapur University of Medical Sciences, Ahvaz, Iran.*; 2 *Department of Nutrition, Health Research Institute, Diabetes Research Center, Ahvaz Jundishapur University of Medical Sciences, Ahvaz, Iran. *; 3 *Department of Biostatistics, School of Health, Ahvaz Jundishapur University of Medical Sciences, Ahvaz, Iran. *

**Keywords:** *Premenstrual syndrome*, *Total antioxidant capacity*, *Serum zinc concentrations*, *Young women*

## Abstract

**Background:**

Premenstrual syndrome (PMS) is one of the important health problems with high incidence in young women. The exact cause of this syndrome is not clear and some theories have been declared from hormonal factors to nutritional disorders.

**Objective:**

We investigated the correlation between serum zinc and antioxidant status with PMS.

**Materials and Methods:**

In this case-control study, forty eight young girls were selected from a total sample of 110 students residing at university dormitories including PMS (n=23) and healthy (n=25) groups based on PMS questionnaire. Dietary intake questionnaire and blood samples were collected from all participants. Serum total antioxidant capacity (TAC) and zinc concentrations were also measured.

**Results:**

Serum TAC and zinc concentrations were lower in PMS patients compared with healthy groups (p<0.01 and p<0.05, respectively). Healthy controls consumed lower servings of hydrogenated oils (p<0.05). There were significant differences in terms of muscle mass between the PMS and healthy groups (p<0.05). Both serum TAC and zinc levels were negatively correlated to PMS scores (r=-0.39, p<0.05 and r= -0.36; p<0.05, respectively).

**Conclusion:**

This study shows that higher TAC and zinc serum levels are associated with lower risk of PMS. PMS cases have more hydrogenated oils than their normal counterparts.

## Introduction

Premenstrual syndrome (PMS) is a mixture of physical, behavioral, and mental symptoms that is felt before the onset of menstrual periods (1). PMS is more common in young women, and WHO has considered PMS as a public health threat in the modern societies (2). PMS comprises two major affective and mental sub groups (3). Approximately 70-90% of women in their fertility period show at least one of the PMS symptoms (4). The prevalence of PMS is 47.8% in the world. Lower and higher rates of PMS are seen in Europe and Asia respectively (5, 6). 

The prevalence of PMS has been reported in different university dormitories of Iran; for example: 96.6% in Arak, 85% in Kerman, 84.62% in Tehran, 54.9% in Bandar Abbas, and 27.8% in Yazd (7, 8). The American College of Obstetricians and Gynecologists has published a set of diagnostic criteria for PMS, such as depression, anxiety, breast tenderness, extremities swelling, headache and abdominal bloating during the five days before menses for three consecutive menstrual cycles (9). However, the exact cause of this syndrome is not completely identified. There are some theories declaring that the genetic vulnerability, sensitivity to hormonal instabilities, and changes in brain neurotransmitters might alter PMS phenomenon (10). Due to high costs and the lack of effectiveness of medications for treatment, many patients tend to practice alternative therapies like dietary supplements, vitamins, and minerals (11).

Several lines of evidence claim that nutrients could affect the mood and behavioral disorders which happen as PMS consequence. Zinc as an essential nutrient for living organisms has a key role in more than 300 enzymes function (12). Low zinc status leads to learning and behavioral deprivation and mood disorders (13). Moreover, zinc has an important function in progesterone binding, prolactin secretion, opiates action, gonadal secretion and regulation of the menstrual cycle (14, 15). Some studies revealed that the level of serum zinc in luteal phase is significantly lower than follicular phase in normal women (16). Similarly, compared to the healthy people, serum levels of zinc in this phase are reported lower in PMS patients (17). 

In healthy people, oxidant and antioxidant activities are in equilibrium. Losing this equilibrium can cause oxidative stress (OS) and it may lead to more than 100 diseases (18). Some studies showed that the level of total antioxidant capacity (TAC) is reduced in PMS patients but others indicated no significant difference in the antioxidant and lipid peroxidation levels between control and PMS patient (19, 20).

Since zinc deficiency has shown a high prevalence, especially in the south of Iran and also, the role of TAC is still controversial, this study was aimed to investigate the association between serum zinc concentrations and body antioxidant status with PMS in young dormitory female students (12).

## Materials and methods

**Participants and procedures**

At first, an analytical cross-sectional study was carried out on 110 medical students (age 21-31 years old) in dormitories of Ahwaz Jundishapur University of Medical Sciences. They were assessed based on PMS questionnaire (Rossignol and Bonnlander scores) in the fall and winter of 2014. Scores <2, 2-16, 17-33, and >33 were considered as normal, mild, moderate, and severe PMS, respectively (21, 22).

Then, 23 students who were identified as moderate to severe PMS were selected as the study group (cases), and 25 students who had not experienced any of premenstrual symptoms were selected as healthy group (controls). Diagnosis of PMS was confirmed by the Daily Symptom Rating Scale (DSR) that included 22 symptoms, each of which was rated based on a 0-3 scale (0=without any symptom, 1=slight, 2=average, and 3=severe). Two groups completed the DSR for 2 consecutive months (23). 

Food intake was measured by a validated 86-item semi-quantitative food frequency questionnaire (FFQ) (24). Blood samples were obtained from each subject in 3^rd^ week (i.e. luteal phase) of menstrual cycle for assessing the serum zinc and TAC concentrations (19). Research protocol was approved by the Medical Ethics Committee at Jundishapur University of Medical Sciences, Ahvaz, Iran. (Ethical approval code: IR.AJUMS.REC.1394.61). 

**Measurement of serum TAC levels, serum zinc concentrations and anthropometric indices**

Serum TAC was measured by ELISA assay (LDN^® ^Labor Diagnostika Nord GmbH, Germany) based on peroxidase reaction and followed by a color reaction of chromogenic substrate tetramethylbenzidine. The change in color was calculated calorimetrically at 450 nm and expressed as “mMol/L” (25). According to kit protocol the expected values were: <1 mMol/L: antioxidative capacity is too low, 1-1.3 mMol/L: is borderline antioxidative capacity, >1.3 mMol/L: is sufficient antioxidative capacity. Serum zinc concentrations were measured by atomic absorption flame method (Chemtech, CTA 3000, England), using wavelength of 213.9 nm and slit width of 0.7 nm. Serum zinc levels <70 μg/dL was regarded as deficiency (26). For anthropometric assessment, percentage of body fat (BF), basal metabolic rate (BMR), muscle mass, weight and viscera fat were obtained by bioelectrical impedance analysis (BIA) method using OMRON device BF-511. Body mass index was also calculated as weight (kg) divided by height (m^2^). The waist and hip size were measured by a non-stretchable meter. 

**Statistical analysis**

In this study, data analysis was done by SPSS software version 22. Kulmogrov-Smirnov test was applied so as to show normal distribution of data. Chi-square test was employed to analyze the qualitative data. Besides, comparisons were made, using independent t-test. In order to assess the linear correlation, Pearson’s coefficient was measured. Statistical significance was considered at p<0.05.

## Results

**Figure 1:**
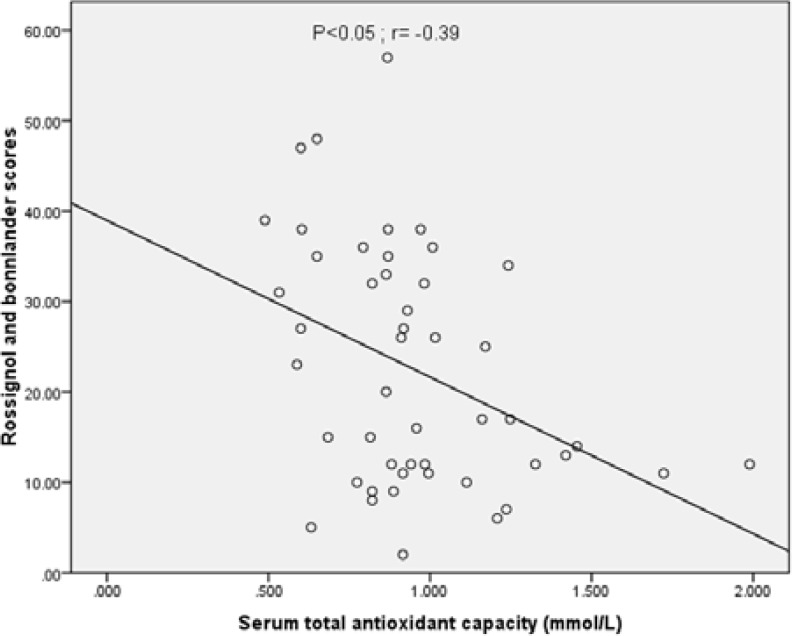
Correlation between PMS scores and serum total antioxidant capacity (µg/deciliter) in PMS patients (r=-0.39; p<0.05).

**Figure 2:**
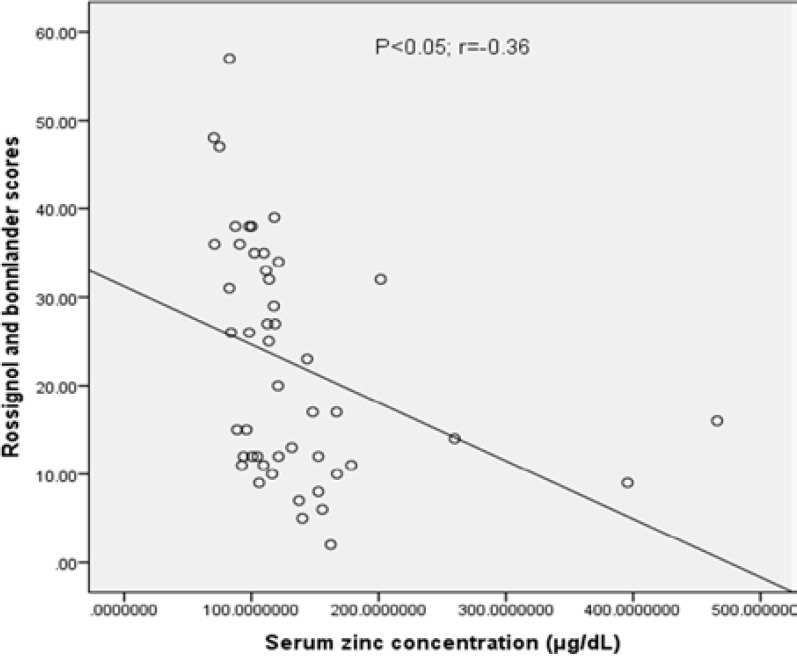
Correlation between PMS scores and serum zinc concentrations (µg/deciliter) in PMS patients (r=-0.36; p<0.05).

As shown in table I, there are no significant differences between the PMS and healthy groups in terms of basic characteristics (age and BMR). However, serum levels of TAC and serum zinc were lower in PMS group (p<0.01). There are significant differences in terms of muscle mass between the PMS and healthy groups (p<0.05, Table II). Consumption of hydrogenated oils was higher in PMS group compared with healthy group (p<0.05, Table III). There were no differences regarding the intake of other food items. Figure 1 indicates a negative linear regression between serum TAC and PMS scores (r= -0.39; p<0.05). The correlation between serum zinc concentrations and PMS scores was also analyzed and a negative linear regression was observed between serum zinc and PMS scores (r=-0.36; p<0.05; Figure 2).

**Table I:** Basic characteristics of the study group

**Criteria **	**PMS (n=23)**	**Healthy (n=25)**	**p-value**
Age (years)	24.17 ± 0.55	23.64 ± 0.60	0.52
TAC ( mmol/L)	0.81 ± 0.041	1.075 ± 0.06	0.00 *
Serum zinc ( μg/dL)	108.20 ± 3.73	153.8 ± 18.77	0.026*
BMR (Kcal)	1297.16 ±20.01	1256.43 ±16.05	0.12
PMS score	33.91 ± 1.80	11.92 ± 1.11	< 0.00***

TAC: total antioxidant capacity

BMR: Basal metabolic rate

* P<0.05;

*** P<0.001 PMS vs. healthy group, Independent t-test was used. Data are shown as mean ± SEM.

**Table II:** Comparison of anthropometric indices between the study groups

**Criteria**	**Healthy (n=25)**	**PMS (n=23)**	**p-value**
BMI	21.19 ± 0.61	22.90 ± 0.67	0.7
Body fat percent (%)	30.24 ± 1.27	34.29 ± 1.39	0.08
Muscle mass (%)	27.24 ± 0.50	25.58 ± 0.48	0.04*
Visceral fat (%)	4.00 ±0.20	3.43 ±0.25	0.08
Waist (cm)	82.78 ±1.84	77.44 ±1.64	0.07
Hip (cm)	98.48 ±1.18	90.56 ± 0.98	0.06

BMI: Body mass index

* P<0.05 PMS vs. healthy group, Independent t-test was used Data are shown as mean ± SEM.

**Table III:** Intake of selected food items as servings in study groups

**Selected food items**	**Servings**	**Healthy (n=25)**	**PMS (n=23)**	**p-value**
Cakes	Daily	1.07 ± 0.15	1.09 ± 0.16	0.9
Snacks	Daily	2.91 ± 0.90	2.19 ± 0.67	0.5
Butter	Weekly	0.90 ± 0.35	1.40 ± 0.40	0.4
Fruits	Daily	1.64 ± 0.16	1.10 ±0.15	0.2
Fresh vegetables	Daily	5.03 ± 0.85	5.03 ± 0.73	0.9
Cooked vegetables	Daily	2.47 ± 0.32	2.84 ± 0.44	0.5
Soft drinks	Weekly	3.51 ± 1.14	7.58± 1.99	0.09
Eggs	Weekly	3.22± 0.54	2.48 ± 0.47	0.3
Red meats	Daily	1.24± 0.16	1.79 ± 0.28	0.1
Chicken	Daily	2.35± 0.43	1.80 ± 0.22	0.3
Fish	Monthly	7.78 ± 2.06	5.90 ± 1.17	0.4
Fats	Daily	0.86 ± 0.24	0.40 ± 0.114	0.06
Refined grains	Daily	5.43 ± 2.85	4.40 ± 1.11	0.7
Whole grains	Daily	9.17± 0.93	8.90 ± 0.89	0.8
Sweets/candies	Weekly	3.88 ± 0.88	6.80 ± 1.36	0.7
Sugars	Daily	3.72 ± 1.35	4.00 ± 1.01	0.8
ice creams	Weekly	5.10 ± 1.37	3.70 ± 1.07	0.4
Tea and coffee	Daily	1.95 ± 0.193	1.70 ± 0.39	0.5
Olive oils	Daily	3.40 ± 1.2	3.70 ± 1.51	0.3
Hydrogenated oils	Daily	0.48 ± 0.28	2.37 ± 0.58	0.00**
Vegetables oils	Daily	7.01 ± 0.61	8.01 ± 0.60	0.3

** P<0.01 PMS vs. healthy group, Independent t-test was used

* Data are shown as mean ± SEM.

## Discussion

Despite several decades of research, the PMS pathophysiology is still unknown. Many supplementary diets and micronutrients potentially influence the improvement of this syndrome through modifications in neurotransmitters and hormones (27). Nevertheless, few studies have focused on the effects of micronutrients on PMS (28, 29). Here, we studied the correlation between serum TAC and zinc levels with PMS.

PMS has a wild spectrum of physical and psychological problems. Major depression and anxiety are the most common psychological problems (23). Several studies evaluated the role of OS in these problems (18). Some researchers showed a high significant increase in malondialdehyde (as OS parameter) concentrations in plasma of the patients with depression (30-32). Other research obtained significantly reduced level of TAC in these patients. A decline in TAC is also associated with increased production of free radicals and decreased levels of antioxidant defenses (33). On the other hand, some researchers agree that hydrogenated oils lead to OS in metabolism of females bodies (33, 34). 

Longhi *et al* found that hydrogenated soybean oils diet was associated with OS in Wistar rats (34). We also showed that women with PMS eat about 5 times more hydrogenated oil servings in their daily meals that may cause increased OS and following by mental disorder. This study also showed that the muscle mass, as an anthropometric indices, was higher in healthy group than in PMS. The muscle mass could be regarded as an index of protein consumption or physical activity. Our data did not show any significant difference between two groups in protein consumption. On the other hand, previous studies revealed that the exercise improved PMS disorders so, increased muscle mass in our normal group might be related to exercise (36, 37). 

To date, there are various discussions on the effects of antioxidants on PMS. In some studies, Kalia *et al* and Balat *et al* investigated the antioxidants levels, such as SOD, glutathione, ceruloplasmin and lipid peroxidation product-MDA in PMS patients (20, 38). They reported no significant differences between the control and PMS patients in antioxidant levels. On the other hand, some studies showed that TAC decreased on the 21^st^ day of menstrual cycle, while oxidant levels increased in PMS patients (19). Also, TAC levels in menstrual and luteal phases were lower than those in follicular phase in PMS cases (39). Today, we know that ovarian hormones perform a basic role in the pathogenesis of PMS and estrogen has a pro-oxidant property that leading to decrease of TAC in these patients (40, 41). However, this study did not check the effect of estrogen in PMS but perhaps this hormone cause to decrease TAC of our PMS group.

In agreement with the previous studies our study indicated that the serum levels of zinc are significantly lower in PMS patients (13, 42, 43). Studies also confirmed that serum zinc levels in luteal phase of a normal menstruation cycle are particularly lower than those in follicular phase (44). Some hypotheses suggested that the reduction in serum zinc during the luteal phase may be due to estrogen levels, enhancement of interleukin-1 or regulating progesterone and prolactin activity (45-47). Das and Chowdhury suggested that zinc is taken up by endometrial tissue during the luteal phase to regulate progesterone and estradiol binding receptors (48).

## Conclusion

Serum TAC and zinc levels in young women with PMS were lower than those of healthy women. The improvement of body antioxidant status through the consumption of rich dietary sources like fresh fruit and vegetables with exercise can improve the syndrome. Hydrogenated oils increase the reactive oxygen species and accordingly, the consumption of the hydrogenated oils could decrease serum TAC levels and subsequently, amplify PMS (31). Finally, the role of healthy nutrition is emphasized in this study. It is suggested that other redox biomarkers and higher number of PMS cases are needed in the future studies.

## Note

***This article extracted from M.Sc. thesis. (Sanaz Fathizadeh)***
