# Corrigendum to “Comparison of serum zinc concentrations and body antioxidant status between young women with premenstrual syndrome and normal controls: A case-control study” [Int J Reprod BioMed 2016; 14: 699-704]

**DOI:** 10.18502/ijrm.v21i7.13925

**Published:** 2023-08-23

**Authors:** Sanaz Fathizadeh, Reza Amani, Mohammad Hossein Haghighizadeh, Razieh Hormozi

The publisher has been informed of an error that occurred on page 699 in which the last authors name must be changed to Razie Hormoznejad. On behalf of the author, the publisher wishes to apologize for this error. The online version of article has been updated on 31 July 2023 and can be found at https://doi.org/10.18502/ijrm.v14i11.699.

